# Airborne dust and high temperatures are risk factors for invasive bacterial disease

**DOI:** 10.1016/j.jaci.2016.04.062

**Published:** 2017-03

**Authors:** Jean-François Jusot, Daniel R. Neill, Elaine M. Waters, Mathieu Bangert, Marisol Collins, Laura Bricio Moreno, Katiellou G. Lawan, Mouhaiminou Moussa Moussa, Emma Dearing, Dean B. Everett, Jean-Marc Collard, Aras Kadioglu

**Affiliations:** a Centre de Recherche Médicale et Sanitaire, Niamey, Niger; b Department of Clinical Infection, Microbiology and Immunology, Institute of Infection & Global Health, University of Liverpool, Liverpool, United Kingdom; c European Public Health Microbiology Training Programme (EUPHEM), European Centre for Disease Prevention and Control (ECDC), Stockholm, Sweden; d Direction de la Météorologie Nationale, Niamey, Niger; e Malawi-Liverpool-Wellcome Trust Clinical Research Programme, University of Malawi College of Medicine, Blantyre, Malawi

**Keywords:** Meningitis, climate, *Neisseria meningitidis*, *Streptococcus pneumoniae*, pollution, dust, A600, Absorbance at 600 nm, CFU, Colony-forming units, OPKA, Opsonophagocytic killing assay, PLY, Pneumolysin

## Abstract

**Background:**

The Sahel region of West Africa has the highest bacterial meningitis attack and case fatality rate in the world. The effect of climatic factors on patterns of invasive respiratory bacterial disease is not well documented.

**Objective:**

We aimed to assess the link between climatic factors and occurrence of invasive respiratory bacterial disease in a Sahel region of Niger.

**Methods:**

We conducted daily disease surveillance and climatic monitoring over an 8-year period between January 1, 2003, and December 31, 2010, in Niamey, Niger, to determine risk factors for bacterial meningitis and invasive bacterial disease. We investigated the mechanistic effects of these factors on *Streptococcus pneumoniae* infection in mice.

**Results:**

High temperatures and low visibility (resulting from high concentrations of airborne dust) were identified as significant risk factors for bacterial meningitis. Dust inhalation or exposure to high temperatures promoted progression of stable asymptomatic pneumococcal nasopharyngeal carriage to pneumonia and invasive disease. Dust exposure significantly reduced phagocyte-mediated bacterial killing, and exposure to high temperatures increased release of the key pneumococcal toxin pneumolysin through increased bacterial autolysis.

**Conclusion:**

Our findings show that climatic factors can have a substantial influence on infectious disease patterns, altering density of pneumococcal nasopharyngeal carriage, reducing phagocytic killing, and resulting in increased inflammation and tissue damage and consequent invasiveness. Climatic surveillance should be used to forecast invasive bacterial disease epidemics, and simple control measures to reduce particulate inhalation might reduce the incidence of invasive bacterial disease in regions of the world exposed to high temperatures and increased airborne dust.

The 1000-km-wide semiarid Sahel region, which lies between the Sahara desert to the north and the Sudanese Savanna to the south, has the highest attack rate (10 per 100,000) and case fatality rates (15%) in the world for bacterial meningitis.^1^, ^2^ This region, which is also known as the meningitis belt, comprises 350 million persons at risk across 21 countries.

Niger, a Sahel country, has a long history of meningitis epidemics, with recent large-scale outbreaks occurring in 2000, 2003, and 2009. *Neisseria meningitidis* serogroups A and X and *Streptococcus pneumoniae* are the main causative agents.^3^, ^4^ Meningitis outbreaks in Niger show strong seasonality, suggesting climatic factors could play a role in disease mechanisms,^5^, ^6^, ^7^, ^8^, ^9^, ^10^ but these studies focus on all-cause meningitis, and little is known about the specific effect of climate on bacterial meningitis.

The dry and dusty Harmattan winds that blow between November and May are a unique defining feature of the West African climate and have been associated with outbreaks of meningitis.^11^ On its passage over the desert, the Harmattan wind picks up fine fractions of Saharan dust particles (mostly particulate matter <10 μm).^11^ The sheer amount of dust in the air can severely limit visibility and sometimes block the sun for several days, which is comparable with a heavy fog. Indeed, the inverse correlation between visibility and particulate matter concentration has been demonstrated in Niger and elsewhere.^12^, ^13^ Dust is thought to have a negative effect on health, increasing morbidity caused by diseases of the upper and lower respiratory tract.^14^

A recent study using a global atmospheric chemistry model has suggested that outdoor air pollution leads to 3.3 million premature deaths per year worldwide, with natural sources of particulate material (predominantly desert dust) responsible for 600,000 (18%) of those deaths.^15^ In large parts of North and East Africa, the Middle East, Central Australia, and Central Asia, natural sources of small particulate material, such as desert dust, make a larger contribution to mortality than more recognized pollution sources, such as industry, traffic, energy, and agriculture. Thus understanding the link between desert dust inhalation and mortality and the climatic factors that influence levels of airborne dust is key to disease control in affected areas.

Long-term forecasting and identification of climatic risk factors would help public health decision makers improve early warning systems and would help the scientific community to identify physiologic factors implicated in the development of invasive diseases. Statistical forecasting models that integrate climatic factors, linking environmental and epidemiologic surveillance, could act as early warning systems of infectious disease epidemics. Here we present findings from a study quantifying, on a daily scale, this link between climate and meningitis in Niamey, Niger. Furthermore, we model these effects *in vivo* by using experimental infection of mice.

## Methods

### Ethics statement

Biological surveillance was performed by the national reference center of the Public Health Ministry of Niger, CERMES (Centre de Recherche Médicale et Sanitaire), which is part of the meningitis national control program.

### Study area and meteorology

The study area was defined as a radius of 50 km around the meteorological station of the international airport of Niamey, Niger, and constituted a homogeneous geographic area for which climatic factors were measured daily. These measures comprise minimal and maximal temperatures, minimal and maximal relative humidity, mean wind speed, mean visibility (defined by the World Meteorology Organization as the maximal distance from which an observer can distinctly see an object on a horizontal plane), and rainfall. Seasons were defined by the National Forecasting Direction (Direction de la Météorologie Nationale) of Niger.

The population of the study area was 1,099,057 for the median year 2006. Cases of meningitis are registered daily, and all cases within the study area confirmed by means of culture, PCR, or both were enrolled between January 1, 2003, and December 31, 2010. Thirty-four health care facilities were involved. Full details can be found in the Methods section in this article's Online Repository at www.jacionline.org.

### Mouse model of *S pneumoniae* infection

All animal experiments were performed at the University of Liverpool in accordance with the Animal Scientific Procedures Act 1986 and with the prior approval of the UK Home Office (PPL 40/3602) and the University of Liverpool ethics committee.

Sex- and age-matched MF1 mice (Charles River, Margate, United Kingdom) were used. Asymptomatic nasopharyngeal carriage was established in mice by means of intranasal infection, as described previously.^16^, ^17^ For particle inhalation experiments, 2 days after infection, mice underwent intranasal administration of 50 mg/mL silicon dioxide (dust; mean particle size, 10 μm; Sigma, Dorset, United Kingdom) or PBS as a control. This was repeated at 4 days after infection, and mice were culled at 7 days after infection or if invasive disease signs (as described by the scheme of Morton and Griffiths^18^) progressed to visible lethargy. For heat exposure experiments, mice were put in a heat box at 40°C for 10 minutes before and for 20 minutes after induction of nasopharyngeal carriage. Control mice were housed at 21°C throughout. The nasopharynx, lungs, brain, and blood were removed and homogenized in PBS before plating on blood agar for assessment of tissue colony-forming units (CFU). Full details can be found in the Methods section in this article's Online Repository.

### Pneumolysin detection ELISA

Sandwich ELISA was performed with mouse anti-pneumolysin (PLY; PLY-4; Abcam, Cambridge, United Kingdom) and rabbit anti-PLY antibody (Abcam). Absorbance at 405 nm was read with a Multiskan Spectrum microplate reader (Thermo Scientific, Waltham, Mass). Full details can be found in the Methods section in this article's Online Repository.

### Opsonophagocytic killing assay

Opsonophagocytic killing assays (OPKAs) were performed, as previously described,^19^ with minor modifications. Briefly, J774 mouse macrophages or HL-60 human neutrophils were incubated with 50 μg/mL silicon dioxide for 1 hour of shaking (175 rpm) before addition of opsonized *S pneumoniae* and complement. CFU values were determined after a further 45 (HL-60) or 60 (J774) minutes of incubation. Full details can be found in the Methods section in this article's Online Repository.

### Measurement of autolytic activity

Triton X-100–induced autolysis assays were performed, as described by Houston.^20^ Full details can be found in the Methods section in this article's Online Repository.

### Hemolytic assay

Overnight cultures of *S pneumoniae* serotype 2 (strain D39) and its isogenic autolysin (LytA)–deficient mutant were subcultured in brain-heart infusion media and incubated at 37°C or 40°C to an absorbance at 600 nm (A600) value of 1.0. Cells were then pelleted, and the supernatant was removed and filter sterilized. Hemolytic activity against sheep red blood cells was measured, as described previously.^21^

### Statistical analysis

A descriptive analysis was performed for the median and interquartile range of the climatic factors, with the range and coefficient of variation according to season. A Mantel-Haenszel χ^2^ test was used to adjust the relative risk for a maximal temperature threshold of greater than 39.5°C on seasons with the statcalc program of Epi Info 6.04 software (Centers for Disease Control and Prevention, Atlanta, Ga).

A generalized additive model with a negative binomial family was used to regress a time series of daily counts of confirmed cases of meningitis with daily changes in climatic factors. Full details can be found in the Methods section in this article's Online Repository. All analyses were performed with R software (R Development Core Team, 2010, version 2.12.0).

Mouse model data were analyzed with GraphPad Prism software (GraphPad Software, La Jolla, Calif) by using ANOVA or a log-rank test with appropriate posttesting. Results with *P* values of less than .05 were considered significant. Data represent means ± SEMs, unless otherwise indicated. Data were assessed for normality by using the D'Agostino-Pearson (omnibus K2) test.

## Results

We conducted daily disease surveillance and climatic monitoring over an 8-year period between January 1, 2003, and December 31, 2010, in Niamey, Niger. Over the 8 years, 893 confirmed cases of bacterial meningitis were recorded within the study site. Epidemics ranged in size from 36 in 2008 to 305 in 2006, with corresponding attack rates of 3.3 to 27.8 10^−5^ (Table I).

**Fig 1:**
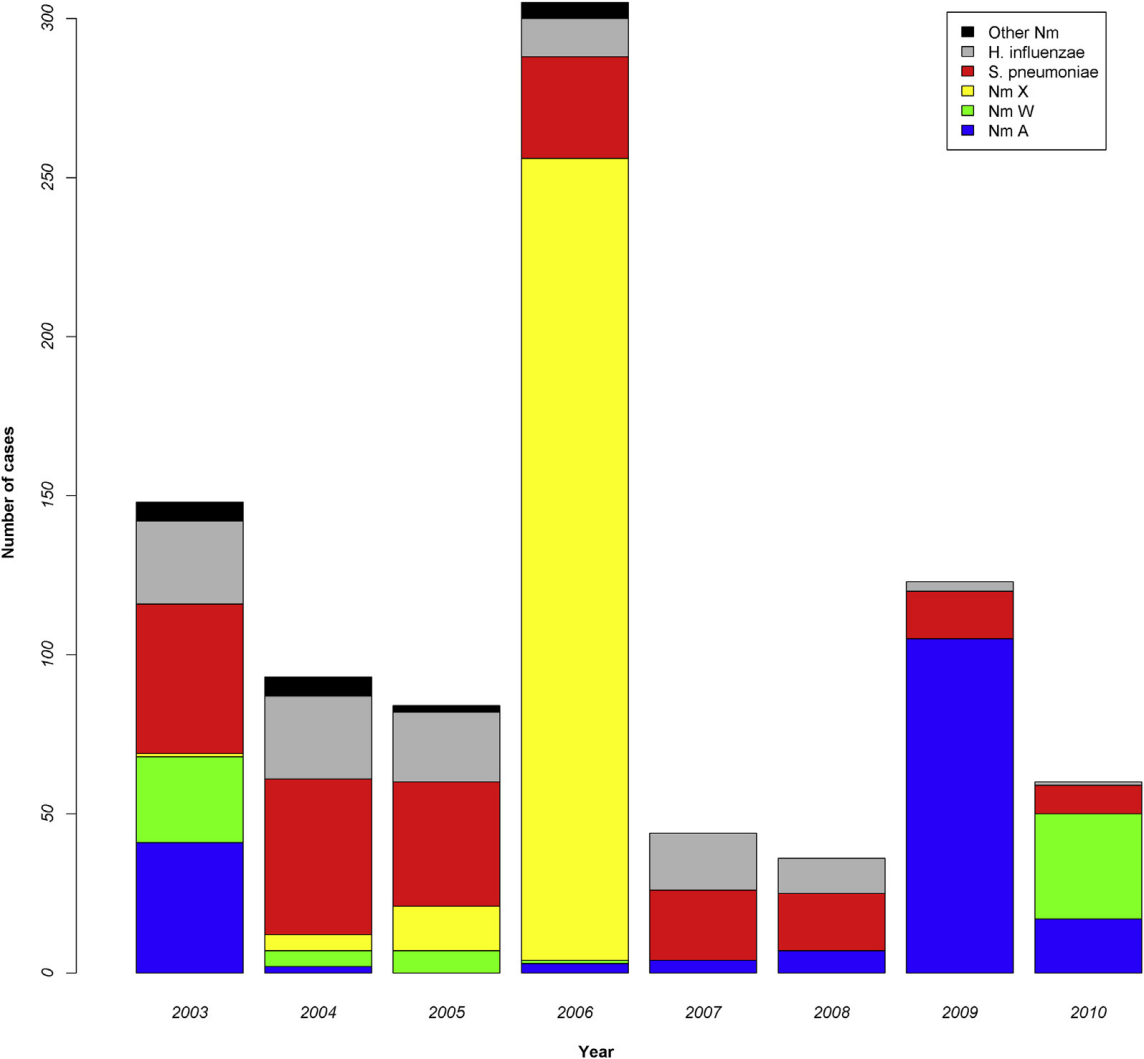
Temporal changes in the causative agent and number of cases of bacterial meningitis in Niamey, Niger. *Nm*, *Neisseria meningitidis*.

Children less than 15 years of age were the most severely affected age group, accounting for 81.7% of cases. *S pneumoniae* was the major cause of meningitis epidemics in 5 of the 8 years and was responsible overall for 25.9% of total cases (Fig 1). *N meningitidis* was the other predominant causative agent, particularly during epidemics, and serogroups X (50.1% of total *N meningitidis* cases) and A (33.0%) were common (Fig 1).

**Fig 2:**
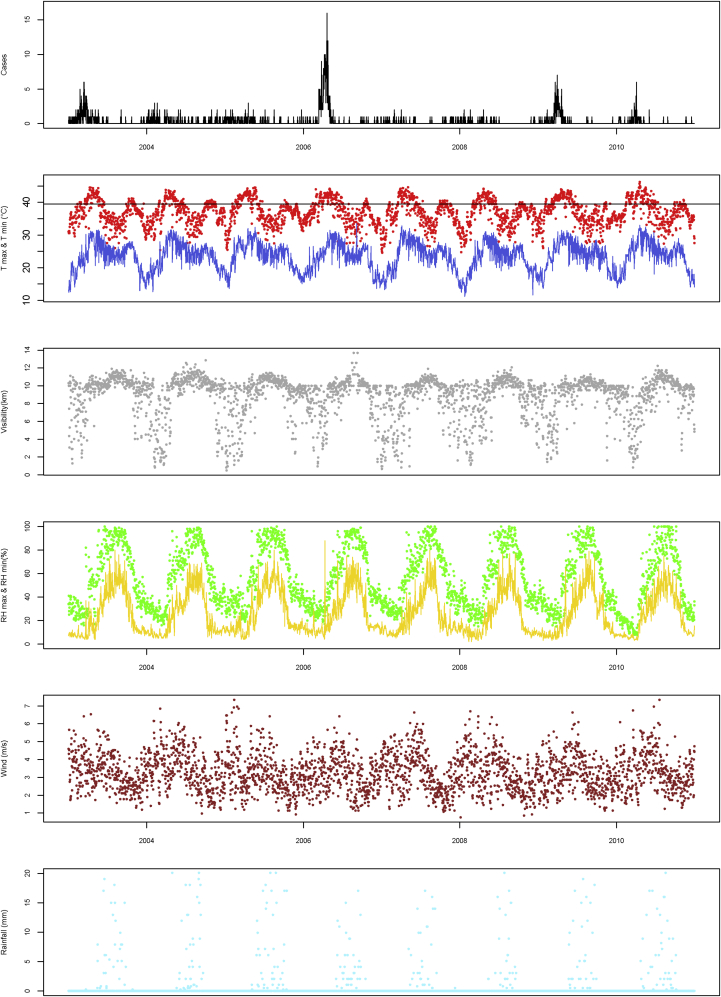
Temporal changes in the number of meningitis cases with climatic factors in Niamey. Visibility is defined as the maximal distance from which an observer can distinctly see an object on a horizontal plane. *RH*, Relative humidity; *T*, temperature.

Climate monitoring demonstrated that all factors other than wind speed displayed strong seasonality (Fig 2 and Table II). High maximal temperatures of greater than 40°C were observed in all seasons, and minimal temperatures of greater than 30°C were recorded during the very hot and rainy seasons. The most striking associations between climatic factors and meningitis cases were increased meningitis cases with increasing maximal temperature, low visibility, and low maximal relative humidity (Fig 2).

**Fig 3:**
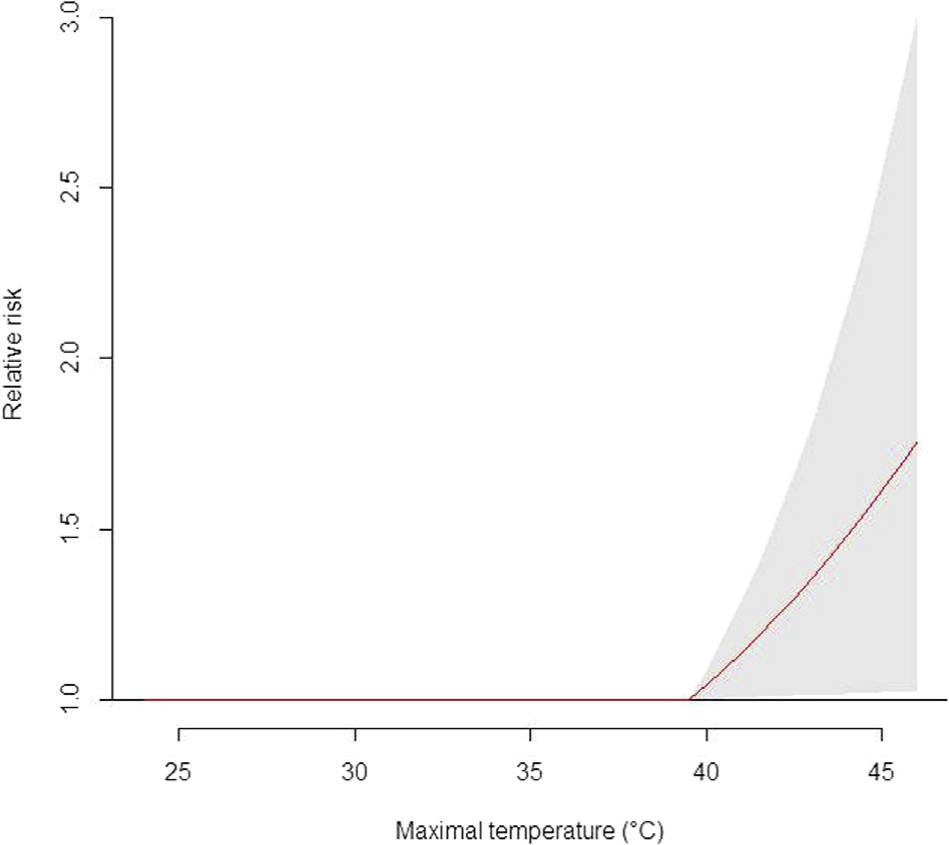
Relative risks for meningitis by maximal temperature from a threshold of 39.5°C. The *gray zone* corresponds to the 95% CI of the risks at greater than a maximal ambient temperature of 39.5°C. A significant effect was observed from a maximal ambient temperature of 39.5°C, and no significant effect was found at less than this value.

The highest numbers of meningitis cases were recorded from a threshold maximum temperature of 39.5°C (β = 0.087, SE = 0.042, *P* = .04), with an excess risk of 9.1% for an increase of 1°C (Fig 3), and this risk could not be explained by seasonal variation in incidence alone (Table III).

An increase in visibility from 0.3 to 5.3 km led to a decrease in the number of meningitis cases (β = −0.49, SE = 0.15, *P* = .001) 34 to 44 days later. Five days after an increase in maximal relative humidity from 38% to 72%, the number of meningitis cases decreased (β = −1.86, SE = 0.69, *P* = .007).

Decreased visibility is predominantly the result of increased airborne dust, and therefore a potential explanation for the association with increased incidence of meningitis is that inhalation of particulate matter during periods of low visibility increases the susceptibility of subjects to invasive bacterial disease. We tested both this hypothesis and the association of temperatures of greater than 39.5°C with invasive bacterial disease in a model of pneumococcal nasopharyngeal carriage. In this model pneumococci stably colonize the naso-oropharynx and carry for long periods with no invasion into the lower respiratory tract and no transmission into the blood.^16^, ^17^ Thus this system models the situation in Niger, where a high proportion of children have asymptomatic nasopharyngeal colonization with potentially pathogenic bacteria, including *S pneumoniae*, *Haemophilus influenzae*, and *N meningitidis*.

**Fig 4:**
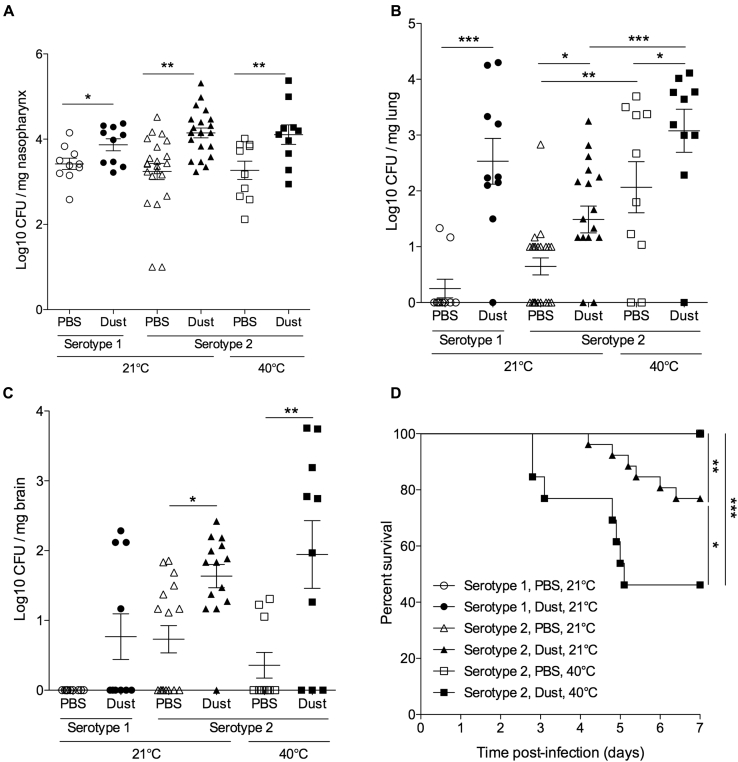
Inhaled dust and exposure to high temperatures increase invasiveness of *S pneumoniae* in mice. Mice were colonized with *S pneumoniae* serotype 2 strain D39 or a Niger serotype 1 meningitis isolate (ST303) and challenged intranasally with dust at 2 and 4 days after colonization. Mice were kept at room temperature (21°C) or 40°C, as indicated for 10 minutes before and 20 minutes after infection and dust/PBS exposure. **A-C,** CFU per milligram of tissue in the nasopharynx (Fig 4, *A*), lungs (Fig 4, *B*), and brain (Fig 4, *C*) at 7 days after infection. **D,** Kaplan-Meier survival curve. *Asterisks* represent significance in 1-way ANOVA with the Dunn posttest (Fig 4, *A-C*) or log-rank analysis (Fig 4, *D*). *P < .05, **P < .01, and ***P < .001.

*S pneumoniae*–colonized mice displayed significantly increased densities of pneumococcal carriage in the naso-oropharynx after dust exposure compared with normal bacterial colonization control mice (Fig 4, *A*). Importantly, this was accompanied by significant invasion of bacteria into the lung and brain after dust exposure (Fig 4, *B* and *C*). This was the case both for mice colonized with the laboratory serotype 2 strain of *S pneumoniae* (D39) and those colonized with a clinical serotype 1 isolate (Fig 4). Serotype 1 *S pneumoniae* isolates were frequently recovered from patients with meningitis in our disease surveillance study (44.8% of *S pneumoniae* cases).

High temperatures also emerged as a significant risk factor for bacterial meningitis, and we sought a direct demonstration of the effect of temperature on invasive bacterial infection. Mice were exposed to temperatures of 40°C (greater than the 39.5°C threshold) for 10 minutes before and 20 minutes after pneumococcal colonization. After heat exposure, pneumococcal numbers in the nasopharynx and brain remained comparable with those in control mice (Fig 4, *A* and *C*), but significantly increased numbers were recovered from the lungs (Fig 4, *B*). This demonstrates that invasive dissemination from the nasopharynx to the lungs occurs after exposure to extreme temperatures. Mice exposed to both dust inhalation and high temperatures had significantly increased bacterial numbers in lung tissue compared with mice exposed to either dust or high temperature alone (Fig 4, *B*).

The combinatorial effect of dust and high temperature was also evident in survival analysis of infected mice (Fig 4, *D*). Visible disease signs and progression to death do not ordinarily occur in the pneumococcal nasopharyngeal carriage model, and this was the case for the serotype 1–colonized mice and the serotype 2–colonized mice that were not exposed to dust (Fig 4, *D*).^16^ However, after dust exposure, 23% of serotype 2–colonized mice had severe invasive disease and had to be culled (Fig 4, *D*). Mortality increased to 54% when dust inhalation was combined with exposure to high temperatures (Fig 4, *D*). All mice that died had significantly increased bacterial loads in their nasopharynx, lungs, brain, and blood compared with survivors (data not shown).

**Fig 5:**
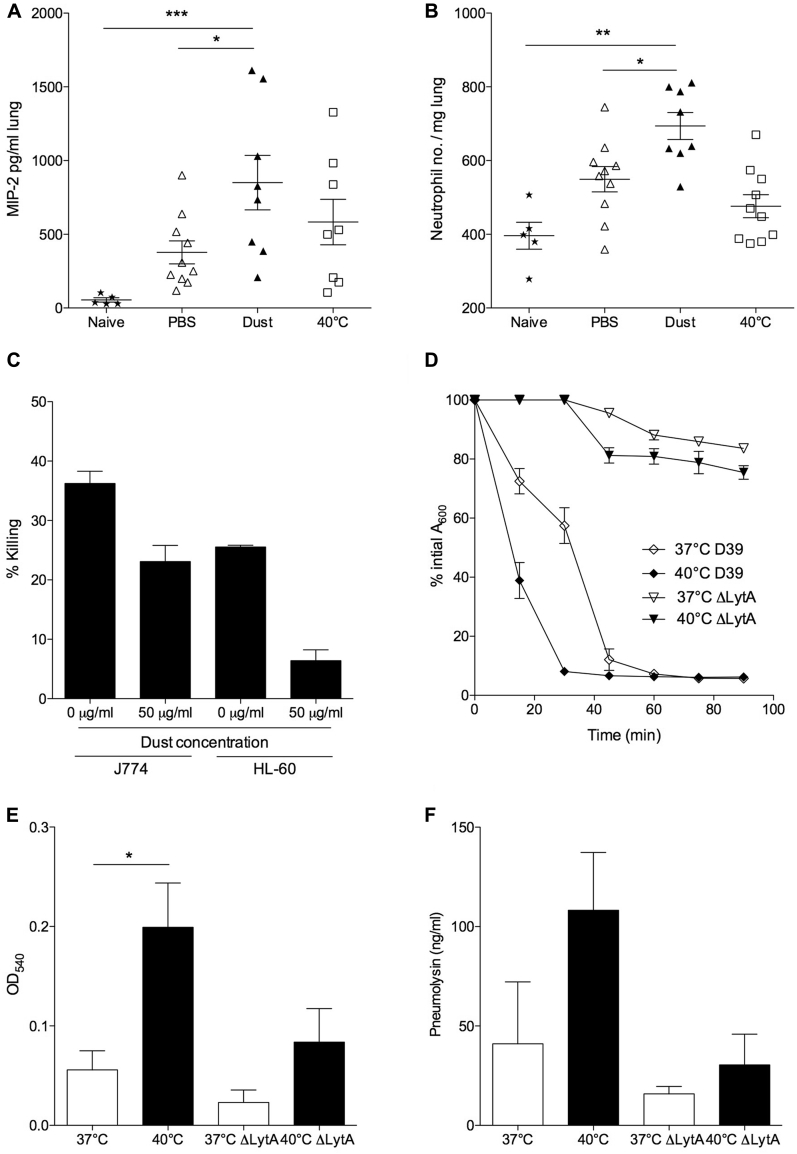
Dust exposure inhibits phagocytosis, and high temperatures induce pneumococcal autolysis and PLY release. **A** and **B,** ELISA quantification of macrophage inflammatory protein 2 (MIP-2; Fig 5, *A*) and flow cytometric determination of neutrophil (Gr-1^high^; Fig 5, *B*) numbers in lungs at day 5 after infection. **C,** Killing (as a percentage of total bacteria added) of D39 by J774 macrophages and HL-60 neutrophils with or without preincubation of phagocytes with dust. **D,** Triton X-100–induced autolysis of serotype 2 (D39) and its autolysin-deficient Δ*lytA* strain grown at either 37°C or 40°C. **E,** Hemolytic activity measured as increased OD after lysis of sheep erythrocytes. **F,** ELISA-calculated PLY concentration in filtered supernatant of D39 and Δ*lytA* grown at either 37°C or 40°C until A600 reached 1.0. Results are representative of 3 independent experiments and are shown as means ± SEMs. *Asterisks* represent significance in 1-way ANOVA with the Dunn posttest. *P < .05, **P < .01, and ***P < .001.

To explore potential mechanisms of dust- or temperature-induced susceptibility to pneumococcal disease, we examined a key component of innate antibacterial defense: phagocytic responses. We observed significantly increased levels of the neutrophil chemoattractant macrophage inflammatory protein 2 (Fig 5, *A*) and increased neutrophil numbers (Fig 5, *B*) in lungs of dust-exposed mice compared with PBS-exposed and naive mice. However, surprisingly, increased infiltration of phagocytic cells did not lead to enhanced bacterial clearance (Fig 4, *A-C*). OPKAs were performed with untreated or dust-exposed neutrophils and macrophages to determine whether dust-exposed phagocytes were impaired in their ability to kill bacteria (Fig 5, *C*). Both macrophage and neutrophil cell lines showed a significantly decreased ability to kill pneumococci after dust exposure (Fig 5, *C*), suggesting that the increased recruitment of phagocytic cells into lungs in dust-exposed mice is ineffective in containment and clearance of pneumococcal infection.

Previous studies in *Staphylococcus aureus* have described temperature-dependent changes in the rate of bacterial autolysis.^22^ We sought to determine whether the enhanced virulence of pneumococci at high temperatures might be due to increased autolysis and thus increased release of the cytosolic toxic PLY. Serotype 2 *S pneumoniae* cultures were grown to OD_600_ 1.0 at 37°C or 40°C before addition of Triton-X to the cultures. Cultures that had been grown at 40°C displayed a markedly increased rate of autolysis compared with those grown at 37°C (Fig 5, *D*). Cell death was significantly reduced in cultures of autolysin-deficient serotype 2 pneumococci grown at either 37°C or 40°C (Fig 5, *D*).

Importantly, increased autolysis was associated with increased release of PLY into the culture medium (Fig 5, *E* and *F*). Supernatant from 40°C cultures induced significantly greater lysis of erythrocytes than supernatant from 37°C cultures (Fig 5, *E*), and 40°C supernatants contained, on average, more than 2-fold higher levels of PLY than 37°C cultures of comparable OD (Fig 5, *F*). Thus increased bacterial lysis and toxin release in the nasopharynx during periods of high temperature might damage the respiratory epithelium, allowing surviving bacteria a route through which to disseminate within the host, and might also lead to lysis of recruited host leukocytes, further impairing antipneumococcal immunity and hampering containment and removal of infection.

## Discussion

We have provided the first quantified risk of the occurrence of meningitis linked to climatic factors, including high temperature, low visibility, and dust. These data demonstrate that environmental exposure to inhaled particulates or extremes of temperature can significantly increase bacterial numbers in the respiratory tract and lead to invasive disease with increased risk of mortality through mechanisms including impaired phagocytic function and increased release of toxins.

The huge epidemic of *N meningitidis* serogroup X meningitis in and around Niamey in 2006 has been reported elsewhere.^23^ Although rare, sporadic epidemics of serogroup X meningitis have occurred previously in Niger.^24^ In all years other than 2006, numbers of meningitis cases caused by *N meningitidis* and *S pneumoniae* were comparable, together accounting for 79% to 96% of cases, with a small but consistent year-on-year contribution from *H influenzae* (1% to 16%).

It is difficult to extrapolate data from a meteorological station to an entire district and therefore impossible to study the link between meningitis and climate without incurring ecological bias. To minimize this bias, daily changes in the count of clinical meningitis cases and climatic factors were obtained throughout the study period (8 years). Furthermore, reinforced microbiological surveillance since 2002 in Niger provides reliable daily counts of biologically confirmed cases of acute bacterial meningitis. Other studies have used data from epidemiologic surveillance based on weekly collection of notifications of suspected meningitis cases at the district level within a meningitis belt country. Consideration should be given to implementation of new models integrating climatic data with high-quality, case-based meningitis surveillance data (based on new World Health Organization guidelines on meningitis outbreak responses) across the African meningitis belt. This could expedite design of effective epidemic control strategies and aid risk management. Dust exposure, for example, could be minimized with simple interventions, such as the use of scarves around the nose and mouth during periods of low visibility.

Saharan dust, carried by the Harmattan, has been shown previously to affect health, particularly by exacerbating asthma and favoring the establishment of respiratory tract infections,^25^, ^26^, ^27^ and is thought to have contributed to meningitis outbreaks in Burkina Faso and Niger.^3^ Previous studies have demonstrated that uptake of particulates by macrophages can disrupt phagocytic bacterial killing,^28^ and we demonstrate here that dust-exposed phagocytes (both macrophages and neutrophils) are functionally impaired. Thus we propose that one mechanism underlying dust-induced disease susceptibility might be that inhalation of dust generates an inflammatory lung condition coupled with impaired phagocytic bacterial clearance, creating an environment conducive to bacterial survival and dissemination to sites, such as the brain.

The ability of inhaled dust to drive up bacterial loads in the nasopharynx is significant because we have recently described how changes in carriage density substantially affect the delicate balance of host immune control in the nasopharynx, driving immune-tolerogenic responses toward damaging proinflammatory responses as bacterial burden increases.^17^ Inhaled dust is likely to trigger inflammatory reactions at the surface of the upper airway mucosal epithelium both through direct abrasion of the respiratory surface and because of its effect on bacterial carriage density. This increased inflammation could induce increased expression of host receptors that act as binding sites for bacteria.^29^ Thus, by triggering local inflammation, inhaled dust can drive colonized bacteria toward a more invasive phenotype.

Set against a backdrop of accelerated climate change, high temperatures could have a strong future effect on the occurrence of bacterial meningitis. Extremes of temperature can cause heat stress in both pathogen and host and thereby favor transition from carrier state in the naso-oropharynx to invasiveness in part through induction of the synthesis of stressor-induced proteins that play a complex role in the phenotypic manifestation of virulence.^30^ Furthermore, at high temperatures, oxidative stress increases, and antioxidants become scarce. Cellular oxidative stress is associated with impairment of host immunity, and immune responsiveness has been found to correlate with levels of antioxidants in plasma in carriers of *N meningitidis*, particularly in children less than 3 years of age.^31^, ^32^

PLY can also be key to the link between high temperatures and invasive bacterial disease. PLY is a key pneumococcal virulence factor and can both promote and dampen inflammation through its ability to induce host cell lysis at high concentrations, as well as inducing a wide range of effects at sublytic concentrations.^29^ PLY-deficient pneumococcal strains are attenuated in virulence in animal disease models, including those of meningitis,^33^ and PLY levels in the cerebrospinal fluid in the setting of meningitis correlate negatively with patient outcomes.^34^

Our data demonstrate an interaction of heat and dust inhalation, whereby mice exposed to both were at significantly increased risk of invasive pneumococcal disease. It might be that the combination of abrasion of the respiratory tract, impaired phagocytosis, and increased release of damaging pathogen toxins creates a “perfect storm” for dissemination of colonized bacteria from the nasopharynx. Alternatively, the effects of particulate inhalation^35^ and high temperatures^36^ on respiration can lead to direct aspiration of precolonized or aerosolized bacteria into the lung. Further mechanistic studies in this area are urgently required to determine how climatic factors contribute to bacterial disease incidence during epidemics.

Collectively, these findings have significant implications for those areas of the world with high bacterial carriage rates coupled with hot climates and high levels of natural pollution. In such settings high levels of atmospheric dust and increased temperatures combine to create a significant risk factor for the development of invasive disease.

**Key messages**

• Temperatures of greater than 39.5°C and increased airborne dust are significant risk factors for invasive pneumococcal diseases, such as pneumonia and meningitis.
• Exposure to high temperatures and inhalation of airborne dust particulates drive progression from stable nasopharyngeal carriage to pneumonia and invasive disease.
• High temperatures and inhaled airborne dust particulates alter the functional activity of host immune cells and promote expression of bacterial virulence factors, leading to increased pathogenicity.
• Limiting exposure to airborne dust in populations with high pneumococcal carriage rates will reduce the risk of invasive disease.

## Figures and Tables

**Table I:** Distribution of meningitis cases per year and according to season and threshold of maximal temperature

	2003	2004	2005	2006	2007	2008	2009	2010	2003-2010∗
Annual cumulative incidence (cases)	148	93	84	305	44	36	123	60	112
Attack rate (cases per 100,000)	13.5	8.5	7.6	27.8	4.0	3.3	11.2	5.5	10.2
Cases during very hot season (%)	77.1	36.3	47.7	93.7	38.6	47.3	87.9	76.7	74.0
Cases when T_max_ ≥39.5°C (%)	35.8	33.3	47.6	89.5	29.5	36.1	82.9	45.0	62.5
No. of days when T_max_ ≥39.5°C	90	89	95	105	97	85	100	113	97

*T*_*max*_, Maximum temperature.

∗ Average of the parameters during the overall period.

**Table II:** Climatic factors suspected to be linked with meningitis by season

Season	T_max_ (°C)	T_min_ (°C)	RH_max_ (%)	RH_min_ (%)	Rainfall (mm)	Wind speed (m · s^−1^)	Visibility (km)
Cold							
Median (IQR)	34.1 (4.8)	17.4 (3.4)	32 (11)	10 (4.3)	0 (0)	6.4 (2.7)	5.4 (2.4)
Range	24.6-41.0	11.2-29.2	13.1-54.0	2-22	0-0	1.4-13.6	0.3-7.1
CV (%)	9.6	14.4	23.1	32.7	NC	31.0	35.6
Very hot							
Median (IQR)	37.5 (4.4)	26.5 (5.6)	33 (29)	12 (14)	0 (0)	6.4 (2.8)	5.4 (0.9)
Range	25.4-46.2	15.5-33.0	7.34-97.0	2-88	0-19	2.2-12.9	0.5-7.1
CV (%)	7.0	14.3	46.8	66.3	13.6	30.6	35.4
Rainy							
Median (IQR)	35 (3.5)	25 (3.5)	86 (16)	46 (17)	0 (0.2)	6.1 (2.8)	6.5 (0.6)
Range	23.7-43.5	18.0-33.5	32-100	10-80	0-7.3	1.8-13.6	3.6-8.5
CV (%)	9.3	9.8	13.8	25.8	2.5	31.2	8.0
Hot							
Median (IQR)	37.5 (3.5)	22 (5.6)	47 (27.2)	15 (11)	0 (0)	4.5 (2.0)	6.0 (2.6)
Range	27.5-41.2	11.6-29.0	20.6-100	5-59.2	0-6.2	1.6-9.8	0.9-8.0
CV (%)	6.8	15.4	32.1	54.6	10.0	30.5	20.6

*CV*, Coefficient of variation; *IQR*, interquartile range; *RH*, relative humidity; *T*, temperature.

**Table III:** Risk for meningitis according to a threshold of maximal temperature of 39.5°C or greater adjusted for season

	No. of days with ≥1 meningitis case when maximal temperature		No. of days with no meningitis case when maximal temperature		RR	95% CI
	≥39.5°C	<39.5°C	≥39.5°C	<39.5°C		
Season						
Rainy	8	52	78	837	1.59	0.78-3.24
Hot	8	36	91	465	1.12	0.54-2.35
Cold	5	101	4	378	2.63	1.43-4.85
Very hot	232	72	348	206	1.54	1.24-1.93
Temperature (crude RR)	253	261	521	1886	2.69	2.31-3.13
Temperature (RR adjusted for season)					1.54	1.26-1.88

*RR*, Relative risk.

## Methods

### Study area and meteorology

The study area was defined as a radius of 50 km around the meteorological station of the international airport of Niamey, Niger. This choice was made in accordance with the National Forecasting Direction (Direction de la Météorologie Nationale) of Niger to obtain a homogeneous geographic area for which climatic factors are measured daily. These measures comprise minimal and maximal temperatures, minimal and maximal relative humidity, mean wind speed, mean visibility (defined by the World Meteorology Organization as the maximal distance from which an observer can distinctly see an object on a horizontal plane), and rainfall. Seasons were defined by the National Forecasting Direction (Direction de la Météorologie Nationale) of Niger.

The population of the study area was 1,099,057 for the median year 2006. Children aged between 0 and 5 years represented 21.9% of the population. Because all cases of meningitis are registered daily, all cases of acute bacterial meningitis confirmed by means of culture or PCR occurring within the study area were enrolled between January 1, 2003, and December 31, 2010. Thirty-four health care facilities were involved in the survey. Almost all the suspected meningitis cases were referred to the national hospital of Niamey (72.3%). The other health care structures in Niamey that participated in the survey were the National Hospital of Lamordé, the La Poudrière Regional Hospital, and 2 health care centers located at the periphery (altogether 13.6% of the meningitis cases). The remaining cases were detected in 1 district hospital, 6 private clinics, and 22 health care centers. Of the total population, 68.2% were vaccinated on April 20, 2009, with a bivalent polysaccharide A/C vaccine after an outbreak involving *N meningitidis* serogroup A (vaccination coverage from 92% to 100% according to the district of Niamey in persons aged 2-30 years). The vaccination coverage was assumed to decrease linearly according to population growth at a daily rate of 0.02%.

### Laboratory testing

Cerebrospinal fluid samples were analyzed by means of PCR at the Niger National Reference Laboratory for bacterial meningitis, where all samples collected in the country are analyzed.^E1^ A confirmed case of acute bacterial meningitis was defined as a first positive culture or PCR for *N meningitidis*, *S pneumoniae*, or *H influenzae*. For *N meningitidis*–positive samples, a slide agglutination with antisera or a second PCR was performed to identify serogroups A, B, C, X, Y, and W. The characteristics of the patient on the epidemiologic form and PCR results were recorded into a database.

### Mouse model of *S pneumoniae* infection

All animal experiments were performed at the University of Liverpool in accordance with the Animal Scientific Procedures Act 1986 and with the prior approval of the UK Home Office (PPL 40/3602) and the University of Liverpool ethics committee.

Sex- and age-matched (8-10 weeks) MF1 mice (Charles River UK) were divided into cages of equal size (usually 3-5 mice) on arrival by animal unit technical staff with no involvement in study design. For a single experiment, weights in all mice were within 2 g (total range for study, 19-24 g) of each other. Investigators were blinded to group allocation, and unblinding was performed after the experiment, when bacterial numbers had been enumerated.

As described previously, asymptomatic nasopharyngeal carriage was established in mice intranasally infected with 1 × 10^5^ CFU of *S pneumoniae* serotype 2 (strain D39) or a Niger serotype 1 strain (ST303) isolated from a child with meningitisE2, E3 in 10 μL of PBS. Infection was performed after achievement of light anesthesia with O_2_/isoflurane. For particle inhalation experiments, 2 days after infection, mice were given intranasal administration of 50 mg/mL silicon dioxide (dust; mean particle size, 10 μm; Sigma) in 10 μL of PBS (10 mice for serotype 1 and 22 mice for D39) or PBS only as a control (10 mice for serotype 1 and 22 mice for D39). This was repeated at 4 days after infection, and mice were culled at 7 days after infection or if invasive disease signs (as described by the scheme of Morton) progressed to visible lethargy.^E4^ For heat exposure experiments, mice were put in a heat box at 40°C for 10 minutes before and 20 minutes after induction of nasopharyngeal carriage (10 mice). Control mice were housed at 21°C throughout (10 mice). The nasopharynx, lungs, brain, and blood were removed and homogenized in PBS before plating on blood agar for assessment of tissue CFU (secondary outcome).

### PLY detection ELISA

Ninety-six-well ELISA microplates (Corning Laboratories, Corning, NY) were coated overnight at 4°C with 1 μg/well mouse anti-PLY (PLY-4) antibody (Abcam). After washing, plates were blocked for 2 hours and washed again, and then 100 μL of bacterial culture supernatant was added for 2 hours. After washing, 1 μg/well rabbit anti-PLY antibody (Abcam) in 100 μL of diluent was added for 2 hours. Plates were washed, and goat anti-rabbit–alkaline phosphatase antibody (Abcam) was added for 30 minutes. After washing, 250 μL/well pNPP color reagent (Sigma-Aldrich, St Louis, Mo) was added for 15 minutes before the reaction was stopped with 50 μL of 3N NaOH. Absorbance at 405 nm was read with a Multiskan Spectrum microplate reader (Thermo Scientific).

### Opsonophagocytic killing assay

OPKAs were performed, as previously described,^E5^ with minor modifications. Briefly, 1 × 10^5^ J774 mouse macrophages or 4 × 10^5^ HL-60 human neutrophils were incubated with 50 μg/mL silicon dioxide (sand; mean particle size, 10 μm; Sigma UK) for 1 hour of shaking (175 rpm) before addition of 1 × 10^3^ opsonized *S pneumoniae* and complement. CFU values were determined after a further 45 minutes (HL-60) or 60 minutes (J774) of incubation. Intravenous immunoglobulin at a final dilution of 1:20 was used as the source of pathogen-specific antibody for opsonization. Wells containing nonopsonized pneumococci were used as controls.

### Measurement of autolytic activity

Triton X-100–induced autolysis assays were performed, as described by Houston et al.^E6^ Overnight cultures of *S pneumoniae* serotype 2 (strain D39) and its isogenic LytA-deficient mutant were subcultured in brain-heart infusion media and incubated at 37°C or 40°C to an A600 of 1.0. Cells were then pelleted and washed twice with PBS and subsequently resuspended in PBS containing 0.02% Triton X-100. The suspensions were then incubated at 37°C or 40°C. A600 readings were taken at 0 minutes and then at 15-minute intervals. Triton X-100–induced autolysis was measured as a percentage of the initial A600 value. Each experiment was repeated 3 times.

### Statistical analysis: General additive model

The general model was written as follows:

Cases = α + *s*(Time) + β.*s*′(Climatic factors) + β_t_.t(Climatic factors) + γ.Factors.

The models were fitted by controlling for long-term trend and seasonality by using a penalized thin-plate regression spline *s* with the mgcv package, which allowed optimization of the numeric method for smoothing and minimized autocorrelation in the residuals. *s′* was defined as spline function, *β* was defined as the coefficient of the variable with nonlinear effects, and *γ* was defined as the coefficient of the linear predictor, such as season, weekdays, holiday, celebration, or vaccination rate. The climatic factors of minimal and maximal temperatures, minimal and maximal relative humidity, rainfall, wind speed, and horizontal visibility were tested as independent covariates in the models. The natural history of meningitis often involves a period of nasopharyngeal carriage before invasion of meninges. The model aimed to explore 2 steps: immediate invasive meningitis after 0 to 5 days of exposure (very short-term effect of environmental exposure) or acute meningitis after a period of carriage (up to 40 days; short-term effect of environmental exposure). A distributed lag model performed with the dlnm package was used, with a natural spline (s′) to estimate the health effect of the climatic factors on the current day and several previous days. The distributed lag model allows the effect of a given day's increase in a given climatic factor to be distributed over several days after its values increase at equally spaced quantiles or quintiles. Several durations, from the daily change of a given climatic factor to that of confirmed meningitis cases, were tested.

A linear threshold parameterization *(t)* was also performed to explore a health effect from given values of climatic factors. It was assumed that a linear effect was observed from a single value of the climatic factor greater than the threshold chosen and null below. The lag dimension was specified from 0 to 5 days. Several thresholds were tested from the median of each climatic factor.

The quality of the adjustment of the models was checked by means of inspection of the sum of partial autocorrelation function (first 30 lags), residuals, and adjustment of predicted data according to observed data. The choice of the final model was based on the Akaike information criterion and adjustment of predicted data according to observed data.^E7^
